# Supplementary material for: Melatonin/Nrf2/NLRP3 Connection in Mouse Heart Mitochondria during Aging
Source: Antioxidants (Basel). 2020 Nov 27;9(12):1187. doi: 10.3390/antiox9121187 (PMC7760557; doi:10.3390/antiox9121187)
Supplement: Supplementary file 1 [file antioxidants-09-01187-s001.zip › antioxidants-999957-images.pdf]

Supplementary Figure S1. Full western data.

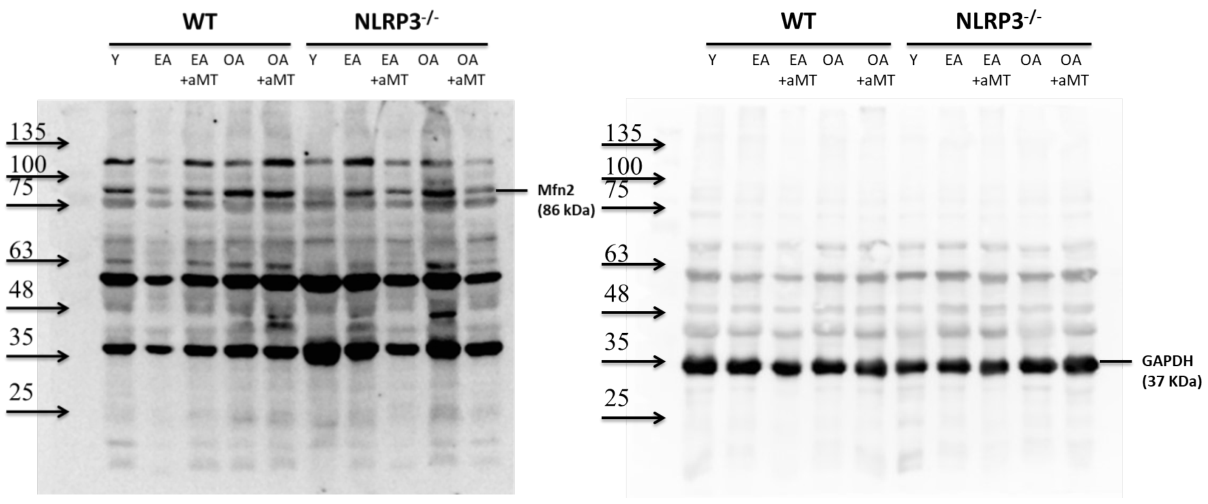

SUPPLEMENTARY FIG.S1. Full scanned Western blots shown in Figure 2A. Cytosolic protein content of Mfn2. Experiments were performed in hearts of young (Y), early-aged (EA), early-aged with melatonin (EA + aMT), old-aged (OA), and old-aged with melatonin (OA + aMT) wild type and NLRP3$^{-/-}$ mice.

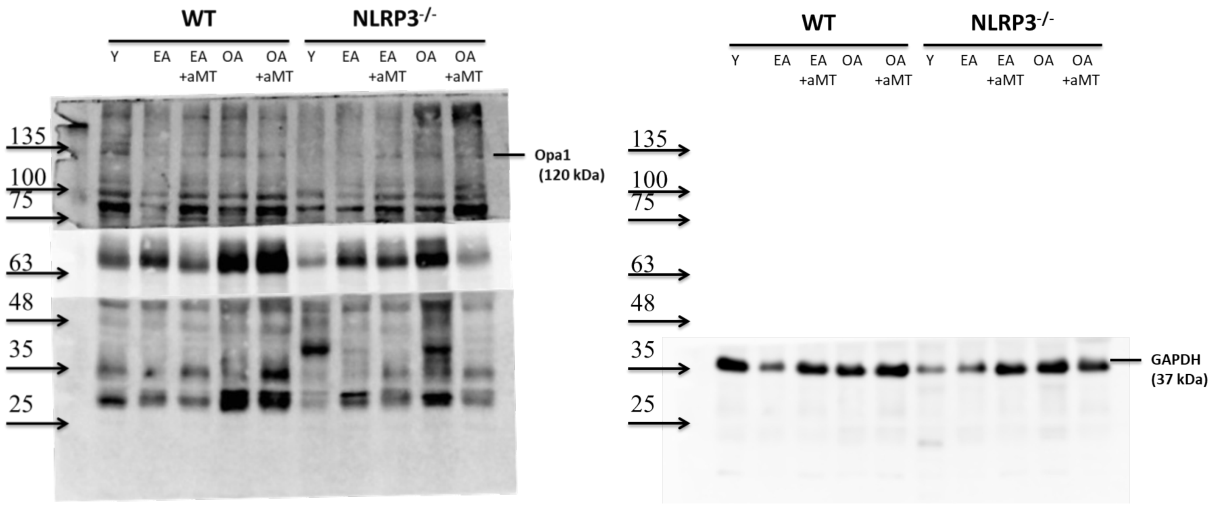

SUPPLEMENTARY FIG.S2. Full scanned Western blots shown in Figure 2B. Cytosolic protein content of Opa1. Experiments were performed in hearts of young (Y), early-aged (EA), early-aged with melatonin (EA + aMT), old-aged (OA), and old-aged with melatonin (OA + aMT) wild type and NLRP3$^{-/-}$ mice.

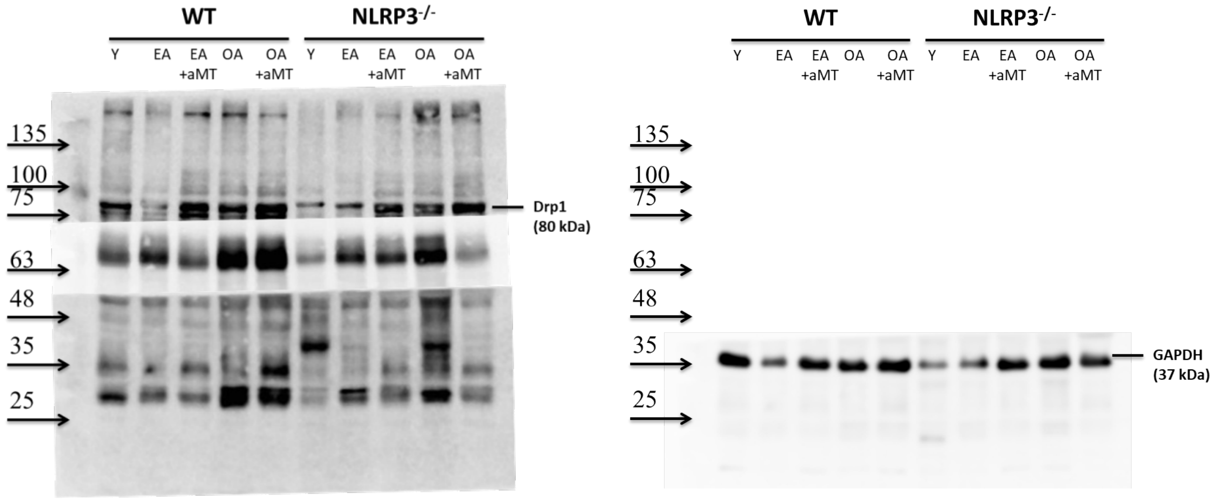

SUPPLEMENTARY FIG.S3. Full scanned Western blots shown in Figure 2C. Cytosolic protein content of Drp1. Experiments were performed in hearts of young (Y), early-aged (EA), early-aged with melatonin (EA + aMT), old-aged (OA), and old-aged with melatonin (OA + aMT) wild type and NLRP3$^{-/-}$ mice.

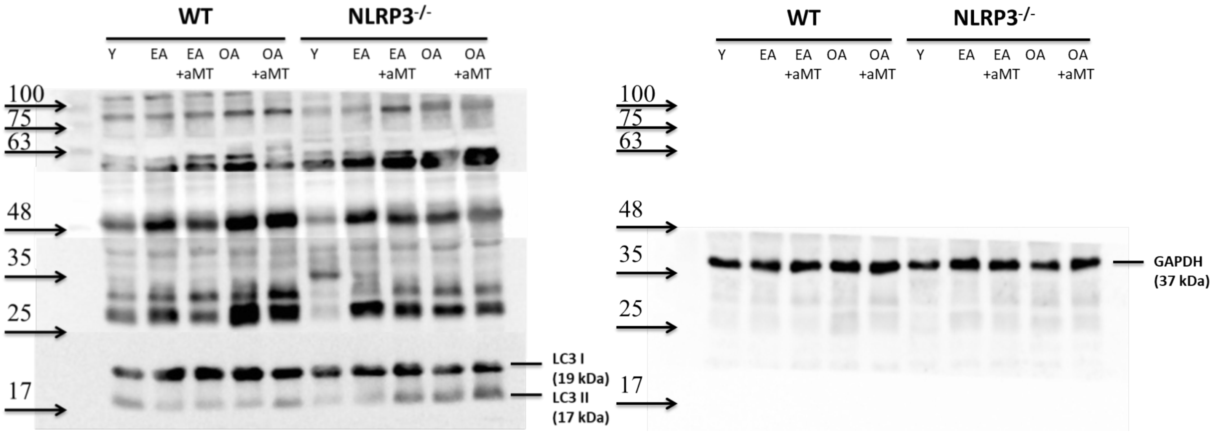

SUPPLEMENTARY FIG.S4. Full scanned Western blots shown in Figures 3A, B. Cytosolic protein content of LC3I and LC3II. Experiments were performed in hearts of young (Y), early-aged (EA), early-aged with melatonin (EA + aMT), old-aged (OA), and old-aged with melatonin (OA + aMT) wild type and NLRP3$^{-/-}$ mice.

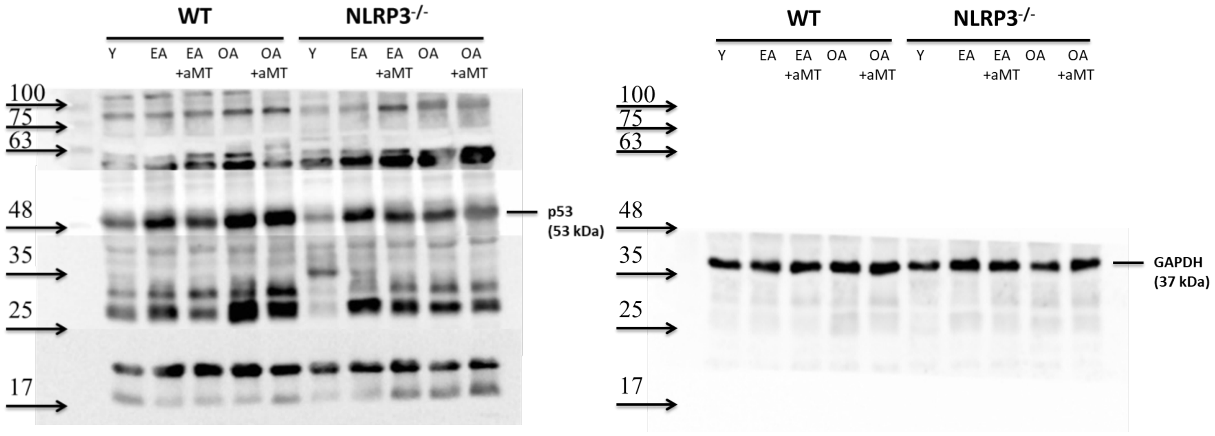

SUPPLEMENTARY FIG.S5. Full scanned Western blots shown in Figure 4A. Cytosolic protein content of p53. Experiments were performed in hearts of young (Y), early-aged (EA), early-aged with melatonin (EA + aMT), old-aged (OA), and old-aged with melatonin (OA + aMT) wild type and NLRP3$^{-/-}$ mice.

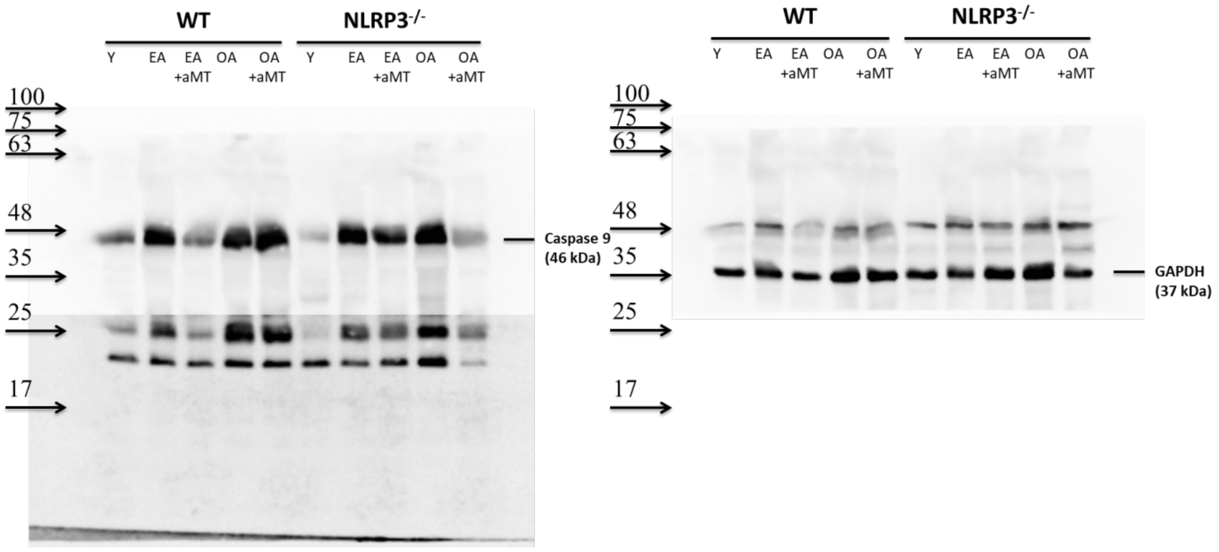

SUPPLEMENTARY FIG.S6. Full scanned Western blots shown in Figure 4B. Cytosolic protein content of Caspase 9. Experiments were performed in hearts of young (Y), early-aged (EA), early-aged with melatonin (EA + aMT), old-aged (OA), and old-aged with melatonin (OA + aMT) wild type and NLRP3$^{-/-}$ mice.

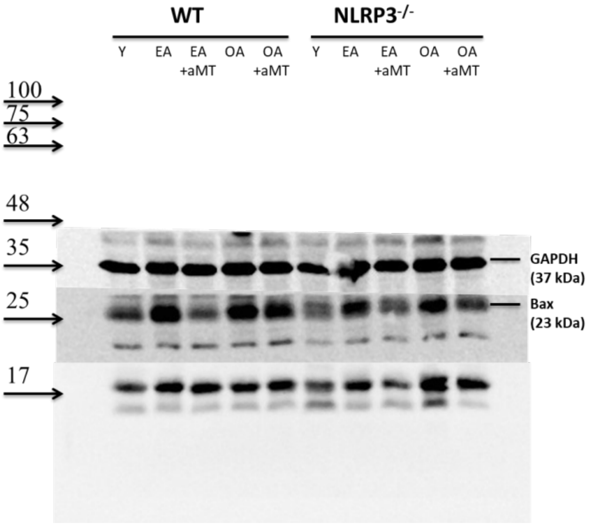

SUPPLEMENTARY FIG.S7. Full scanned Western blots shown in Figure 4C. Cytosolic protein content of Bax. Experiments were performed in hearts of young (Y), early-aged (EA), early-aged with melatonin (EA + aMT), old-aged (OA), and old-aged with melatonin (OA + aMT) wild type and NLRP3$^{-/-}$ mice.

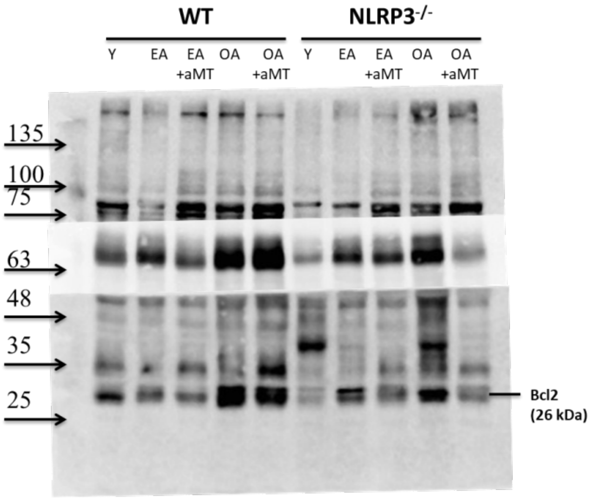

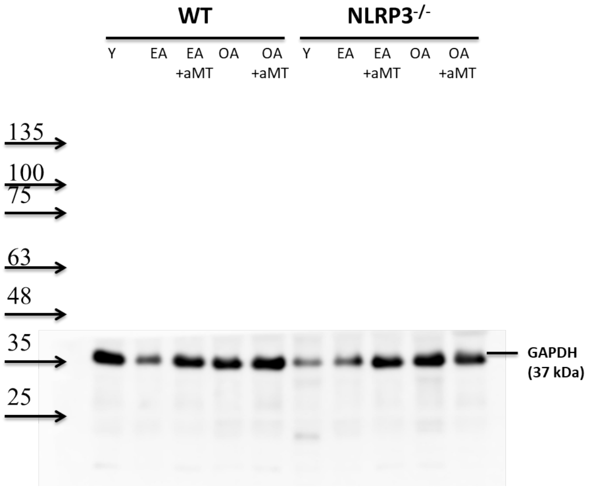

SUPPLEMENTARY FIG.S8. Full scanned Western blots shown in Figure 4D. Cytosolic protein content of Bcl2. Experiments were performed in hearts of young (Y), early-aged (EA), early-aged with melatonin (EA + aMT), old-aged (OA), and old-aged with melatonin (OA + aMT) wild type and NLRP3$^{-/-}$ mice.

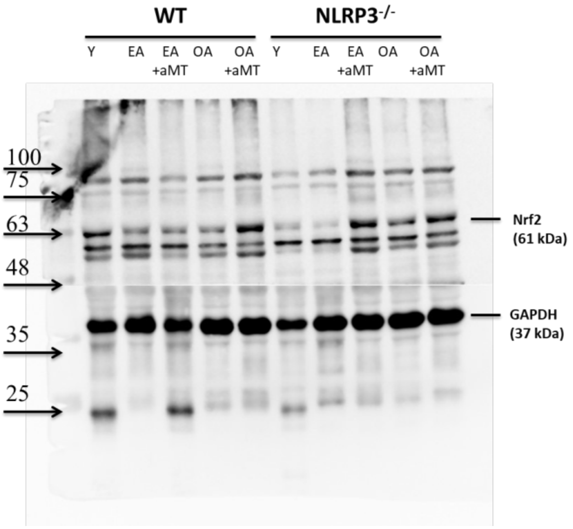

SUPPLEMENTARY FIG.S9. Full scanned Western blots shown in Figure 5A. Cytosolic protein content of Nrf2. Experiments were performed in hearts of young (Y), early-aged (EA), early-aged with melatonin (EA + aMT), old-aged (OA), and old-aged with melatonin (OA + aMT) wild type and NLRP3-/- mice.

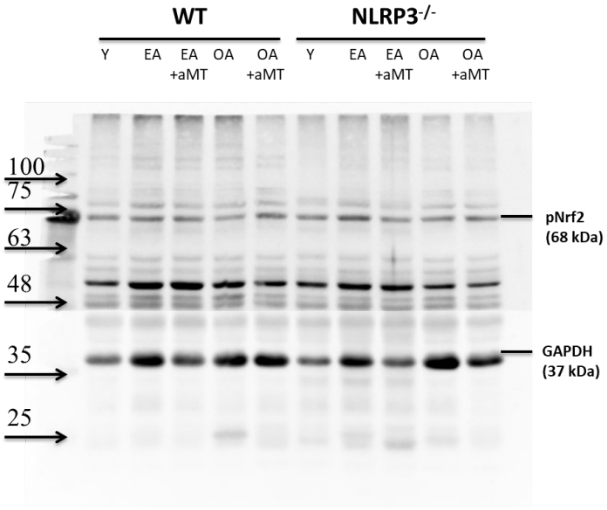

SUPPLEMENTARY FIG.S10. Full scanned Western blots shown in Figures 5B. Cytosolic protein content of pNrf2. Experiments were performed in hearts of young (Y), early-aged (EA), early-aged with melatonin (EA + aMT), old-aged (OA), and old-aged with melatonin (OA + aMT) wild type and NLRP3-/- mice.

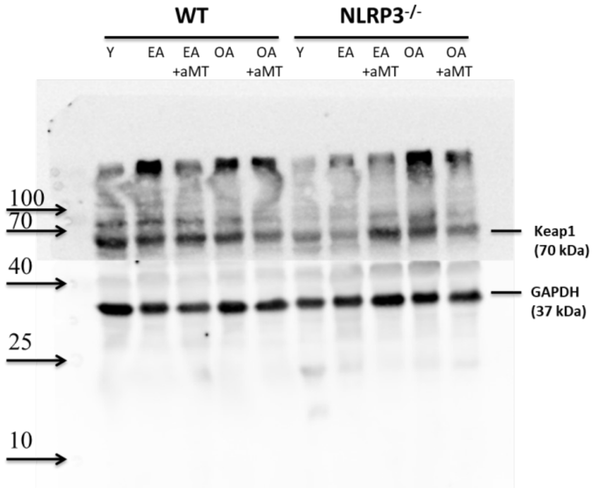

SUPPLEMENTARY FIG.S11. Full scanned Western blots shown in Figure 5C. Cytosolic protein content of Keap1. Experiments were performed in hearts of young (Y), early-aged (EA), early-aged with melatonin (EA + aMT), old-aged (OA), and old-aged with melatonin (OA + aMT) wild type and NLRP3$^{-/-}$ mice.

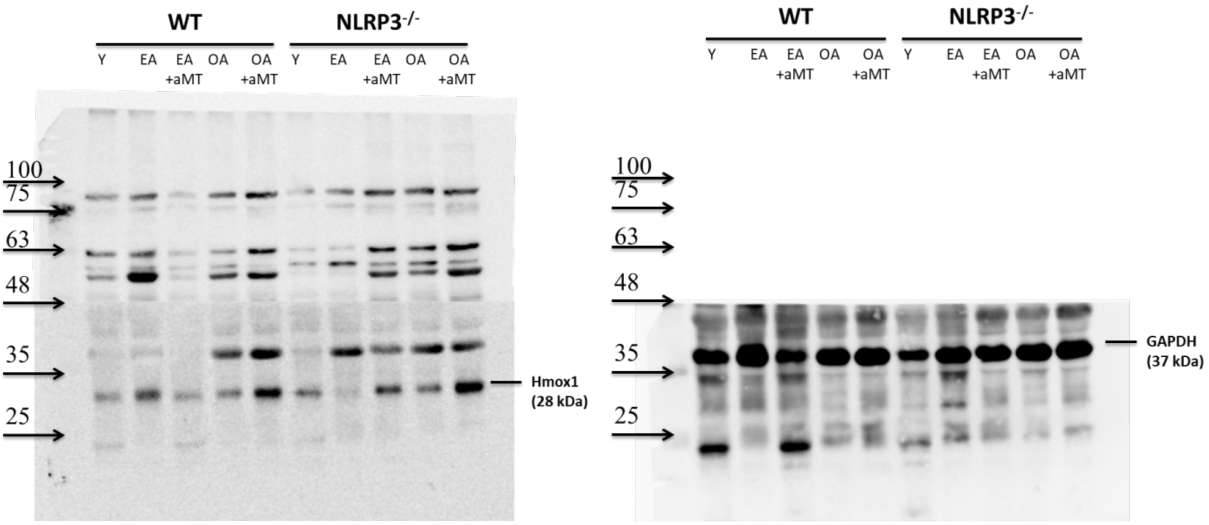

SUPPLEMENTARY FIG.S12. Full scanned Western blots shown in Figures 5D. Cytosolic protein content of Hmox. Experiments were performed in hearts of young (Y), early-aged (EA), early-aged with melatonin (EA + aMT), old-aged (OA), and old-aged with melatonin (OA + aMT) wild type and NLRP3$^{-/-}$ mice.

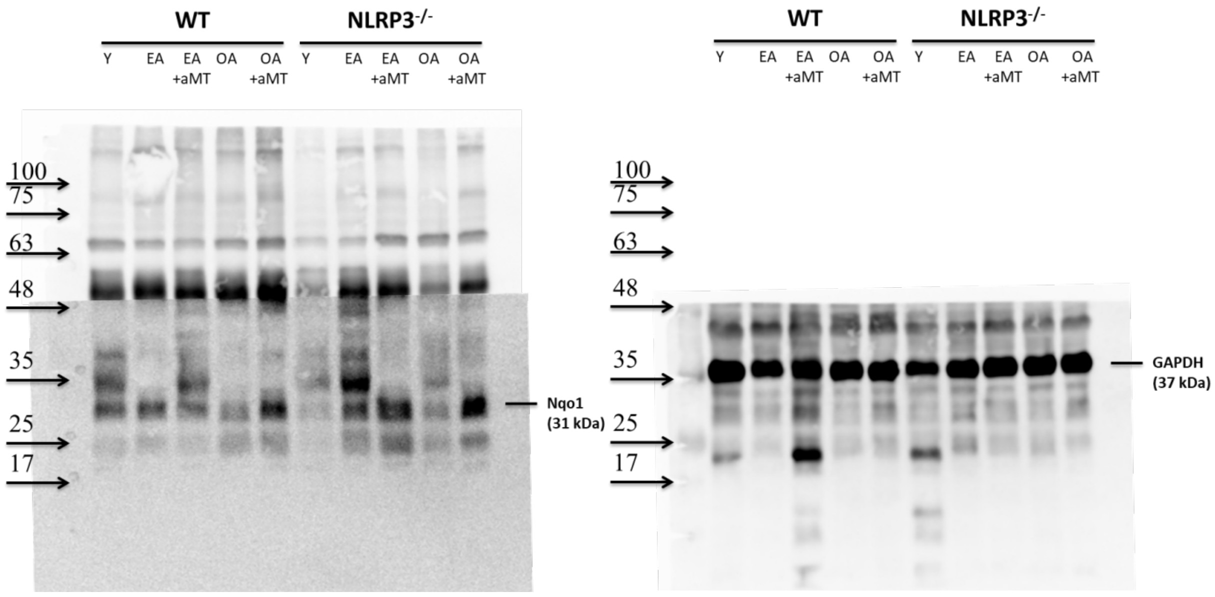

SUPPLEMENTARY FIG.S13. Full scanned Western blots shown in Figure 5E. Cytosolic protein content of Nqo1. Experiments were performed in hearts of young (Y), early-aged (EA), early-aged with melatonin (EA + aMT), old-aged (OA), and old-aged with melatonin (OA + aMT) wild type and NLRP3$^{-/-}$ mice.

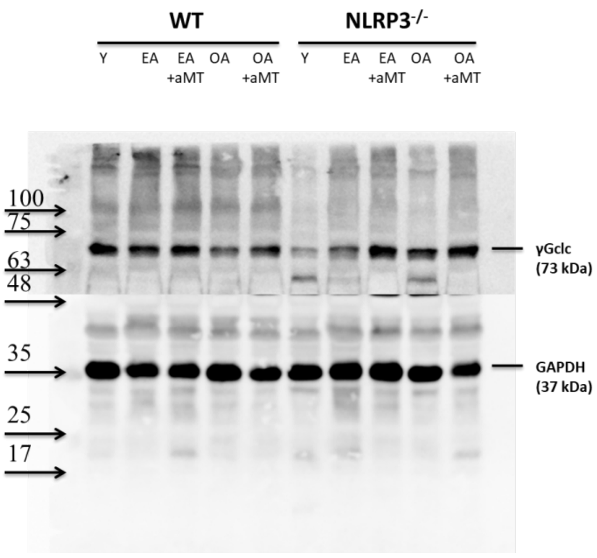

SUPPLEMENTARY FIG.S14. Full scanned Western blots shown in Figures 5F. Cytosolic protein content of γGclc. Experiments were performed in hearts of young (Y), early-aged (EA), early-aged with melatonin (EA + aMT), old-aged (OA), and old-aged with melatonin (OA + aMT) wild type and NLRP3$^{-/-}$ mice.
