# Supplementary material for: Melatonin/Nrf2/NLRP3 Connection in Mouse Heart Mitochondria during Aging
Source: Antioxidants (Basel). 2020 Nov 27;9(12):1187. doi: 10.3390/antiox9121187 (PMC7760557; doi:10.3390/antiox9121187)
Supplement: Supplementary file 1 [file antioxidants-09-01187-s001.zip › antioxidants-999957-tables.pdf]

**Supplementary Table S1.** List of primary antibodies used in western blot analysis.

| Antibody | Reference | Company |
|---|---|---|
| *Anti-Bax* | sc-7480 | Santa Cruz Biotechnology (Heidelberg, Germany) |
| *Anti-Bcl2* | sc-7382 | |
| *Anti-Casp9* | sc-56076 | |
| *Anti-p53* | sc-126 | |
| *Anti-GAPDH* | sc-166574 | |
| *Anti-Nrf2* | sc-722 | |
| *Anti-Nqo1* | sc-32793 | |
| *Anti-γ-Gclc* | sc-390811 | |
| *Anti-Keap1* | 10503-2-AP | Proteintech (Manchester, United Kingdom) |
| *Anti-Hmox1* | 70081s | Cell Signaling Technology (Leiden, The Netherlands) |
| *Anti-pNrf2 (Ser40)* | bs-2013R | Bioss Antibodies (Woburn, MA, USA) |
| *Anti-LC3* | NB100-2220 | Novus Biologicals (Centennial, CO, USA) |
| *Anti-Opa1* | CPA3687 | Quimigen (Madrid, Spain) |
| *Anti-Drp1* | PA5-43802 | Fisher Scientific (Madrid, Spain) |
| *Anti-Mfn2* | TA344104 | OriGene Technologies (Rockville, MD, USA) |

*Bax: BCL2 associated X, apoptosis regulator; Bcl2: BCL2 apoptosis regulator; Casp9: caspase 9; p53: transformation related protein 53; GAPDH: glyceraldehyde 3-phosphate dehydrogenase; Nrf2: nuclear factor, erythroid derived 2, like 2; Nqo1: NAD(P)H dehydrogenase, quinone 1; γ-Gclc: γ-L-glutamate-L-cysteine ligase, catalytic subunit; Keap1: kelch-like ECH-associated protein 1; Hmox1: heme oxygenase 1; pNrf2: phosphorilated Nrf2 (Ser40); LC3: microtubule-associated protein 1 light chain 3; Opa1: OPA1, mitochondrial dynamin like GTPase; Drp1: dynamin related protein 1; Mfn2: mitofusin 2.*
